# A Clinical Introduction to the Practice of Auscultation and Other Modes of Physical Diagnosis in Diseases of the Lungs and Heart

**Published:** 1854-10

**Authors:** 


Art. IV.—*A Clinical Introduction to the Practice of Auscultation and other modes of Physical Diagnosis in Diseases of the Lungs and Heart.* By H. M. Hughes, M. D., Fellow of the Royal College of Physicians; Assistant Physician to Guys Hospital, etc. Second American from the second and revised English edition. Philadelphia: Blanchard & Lea. 1854. 12mo., pp. 304.

Dr. Hughes truly remarks, that it cannot be necessary at the present day to insist upon the advantages of auscultation and percussion to the practical physician, and to every legitimate practitioner of the healing art. The race of physicians who once discredited these modes of diagnosis has well nigh passed away. There may be those among their successors, and we fear there are many, who still neglect them, but we doubt whether there are any who would have the hardihood to deny their utility. All that great improvement in the diagnosis of diseases of the chest made in the last forty years, is attributable to the introduction of these methods. But they require time, patience, assiduity, and perseverance in the student. Proficiency in their use is not to be acquired in a day, much less by intuition. A prolonged education is necessary to their skillful employment, but the facility that may be attained without such thorough training is useful, and the work of Dr. Hughes is an excellent manual for the student and practitioner who may not enjoy opportunities of applying them in hospital practice.

Dr. Hughes begins with giving all the directions necessary to the application of the physical means of diagnosis. Among others he lays down that—

"It is essential to the successful prosecution of auscultation and percussion that their value should be fully, but at the same time fairly, estimated. If the individual employing them be influenced merely by fashion, and labor under the misconception that they are in reality of little importance to the formation of a correct diagnosis, it is highly probable that, in his hands at least, they will prove so. If, on the other hand, the student conceive that by their use he will be enabled, in every case, to determine the exact condition of a diseased organ, he will be most assuredly liable to much disappointment. They should never be, and there is no valid reason why they ever should be, employed to the exclusion of the other modes of investigation. When the history, and the general or constitutional symptoms of disease, have been inquired into with the same minuteness, and interest as in other cases; then, but usually not till then, should percussion and auscultation be employed, or then, at any rate, and not till then, should any deductions be made from the indications afforded by the physical signs. To a neglect, or comparative disregard of the history, and ordinary symptoms of disease, and a too implicit confidence in their highly important art, it is probable that some of the errors of even accomplished auscultators are to be attributed. There is, however, I repeat, no valid reason why the auscultator should not use with equal diligence, and be equally skilled in the employment of all other modes of investigation, as the individuals who discard the stethoscope, and make no use of the ear in forming their diagnosis, and why he should not enjoy the advantage of his art *in addition* to all those possessed by others. Let, then, auscultation and percussion be regarded as ancillary only to other, or rather as two among many, modes of investigating disease; as two stout and strong additional strings to our bow; albeit the information they afford is, in some cases, equal, if not superior, to that derived from all other modes combined."

Many truly valuable works have, within a few years, been prepared with a view to facilitating the study of auscultation and percussion in diseases of the chest, but we are not sure that one has been offered to the public containing more ample instruction in the same compass than this little volume, and our belief is that such readers as are in quest of a manual on the subject could not do better than to avail themselves of the guidance here tendered by Dr. Hughes.

